# Assessment of marketing mix associated with consumer's purchase intention of dairy products in Bangladesh: Application of an extended theory of planned behavior

**DOI:** 10.1016/j.heliyon.2023.e16657

**Published:** 2023-05-30

**Authors:** Md. Shaikh Farid, Alessio Cavicchi, Md. Mostafizur Rahman, Swarup Barua, Dilshad Zahan Ethen, Fardous Ara Happy, Md. Rasheduzzaman, Dabasis Sharma, Mohammad Jahangir Alam

**Affiliations:** a Department of Agricultural Marketing and Business Management, Sylhet Agricultural University, Bangladesh; b Department of Agriculture, Food and Environment, University of Pisa, Italy; c Department of Agribusiness and Marketing, Bangladesh Agricultural University, Bangladesh; d Department of Agricultural Economics, Khulna Agricultural University, Bangladesh; e Bangladesh Agricultural Research Institute (BARI), Bangladesh

**Keywords:** Marketing mix, Purchase intention, Dairy products, Bangladeshi consumers, TPB, SEM

## Abstract

Despite persistent challenges, Bangladesh's dairy sector has been noticeable for a few decades. Although agriculture is the major contributor to GDP, dairy farming may play a crucial role in the economy by creating jobs, ensuring food security, and boosting the protein content of people's diets. This research aims to identify the direct and indirect factors influencing dairy product purchase intention amongst Bangladeshi consumers. Data were collected online using Google forms, and the convenience sampling technique was used to reach the consumers. The total sample size was 310. The collected data were analyzed using descriptive and multivariate techniques. Structural Equation Modeling results reveal that marketing mix and attitude are statistically significant with the intention to purchase dairy products. Also, the marketing mix influences the consumers' attitudes, subjective norms, and perceived behavioral control. However, there is no significant association between perceived behavioral control and subjective norm on intention to purchase. The findings suggest developing better products, ensuring reasonable pricing, performing promotional strategies, and proper placement to attract and increase consumers' intention to purchase dairy products.

## Highlights

• Dairy farming can provide jobs, food security, and boost protein intake.
• This study used the Extended Theory of Planned Behavior to identify the purchase intentions of dairy consumers.
• The marketing mix and attitude influenced dairy buyers significantly.
• The study found no significant correlation between perceived behavioral control and subjective norm on purchasing intentions.
• Promotional campaign and product's availability in convenient locations might increase purchase intentions.

## 1 Introduction

The United Nations Sustainable Development Goal emphasizes good health and well-being, making food safety and health the most pressing concerns today [1]. The quantity of consumption of dairy products varies across the world. Dairy products have enormous health benefits because dairy products are a multi-component blend considering nutritional values and fundamental nutrients for all age classes [2]. Cow's milk and high-fat milk products provide good saturated fat, which can be used as an energy source to help the body gain muscle mass. The demand for fresh milk and dairy products worldwide has increased significantly in the last decade. Consumption has also risen in other developing countries like Eastern and Middle Eastern Asia and Africa [3]. According to the Department of Livestock Services, Bangladesh's milk production stood at 10.68 million metric tons during 2019–20. The World Health Organization (WHO) advises a minimum daily dairy milk consumption of 250 ml per person. However, annual milk deficiency is around 4.81 million metric tons compared to the total demand of 15.49 million tons [4].

Understanding the consumer is challenging since purchasing is a decision process involving different factors like upward shifting the consumer demand for dairy products in developing countries like Bangladesh. The rise of the middle class is due to rapid urbanization and the spread of modern retail markets for high-end products and safety standards [5]. Additionally, the government takes initiatives to improve the population's nutritional status, infrastructural growth, and milk imports for milk deficit countries like Bangladesh to fulfill the demand. These factors increased food consumption expenditures, especially on proteins and fats, for rural households in developing countries, increasing the demand for better quality and safety of dairy products [6,7]. Therefore, it is essential to undertake consumer behavior research to comprehend what motivates consumers to purchase products and what needs to be done to enhance consumer engagement.

The realization of the consumer buying decision process is a precondition for gaining market share for any organization's success and a critical factor in suggesting policy recommendations. In light of market dynamics and macro-dynamics (political, demographic, nutritional, etc.), it is essential to comprehend the most influential consumer factors. Over the years, dairy product purchasing intentions have been researched in developed and developing countries [8,9]. However, to our knowledge, no empirical research has been conducted in Bangladesh integrating the marketing mix (product, price, place, and promotion) with the ‘Theory of Planned Behavior’ to explain consumers' purchasing intentions. Thus, this study aims to fill this gap by identifying the direct and indirect factors influencing consumers' decisions to buy dairy products. This paper seeks the association of the marketing mix with consumers' purchase intention of dairy products in Bangladesh. This paper also examined which marketing mix impacts the purchase intention of dairy products in Bangladesh.

The paper is divided into six sections, starting with background information on dairy sectors and briefly describing the Theory of Planned behavior in section two. The third section deals with the methodology and analytical techniques used for the study. The results of the study are presented in the fourth section. The fifth section includes a discussion of the findings with prior empirical knowledge. Last, a conclusion highlights the significant points with policy implications.

## 2 Theory of planned behavior

The Theory of planned behavior (TPB) proposes three conceptually distinct drivers of intention: attitude toward the behavior, subjective norm, and perceived behavioral control [10]. Attitude toward behavior is how a person evaluates or judges it. Subjective norms define the pressures from society to do or not do something. Behavioral control shows if a product is easy for a consumer or hard or impossible to use [11]. Intention refers to individual willingness or motivation to engage in a particular behavior. Various studies have demonstrated that attitude is a crucial precursor of behavioral intent [[12], [13], [14]]. Subjective norm is the vital determinant that influences consumer behavior. The empirical investigation demonstrated a significant correlation between subjective norms and purchasing intent. Perceived behavioral control (PBC) is among the most influential consumer behavior factors. Perceived Behavioral Control refers to "people's perception of the ease or difficulty of performing the behavior of interest” [14]. This aspect has something to do with resources, opportunities, and barriers. In addition, additional resources to act and fewer obstacles would increase the motivation to work. Research suggests an indirect relationship between purchasing behavior and customer intention. The TPB effectively explores consumers' underlying motives, especially for drinking, use of contraceptives, smoking, etc. There are various behavioral models to determine the relationship between purchasing behavior and consumer intention [15]. TPB, Theory of Reasoned Action, traditional attitude-behavior, and behavioral perspective models are the most prevalent theoretical models in consumer behavior. Researchers can use these models and theories to figure out and investigate consumer behavior related to food intake and product purchases [16,17].

### 2.1 Attitude toward behavior

The degree of an individual's positive or negative feelings toward any object or toward the intention of performing any particular behavior is called attitude. As per the TPB, TRA, and TAM, attitude is one of the significant predictors of consumers' behavioral intention [14]. Attitudes can predict an individual's behavioral intentions towards purchasing any product [18]. In marketing, a consumer can have different attitudes towards the same commodity in various circumstances, as attitudes can perform favorable and unfavorable behavior [19]. Previous studies have found a positive relationship between attitude toward behavior and intention to purchase dairy products [20,21]. Therefore, those empirically verified positive influences of attitude on purchase intention of dairy products enforce the study to adopt the below hypothesis.

H1 Attitude shows a positive relationship with purchase intention of dairy products.

### 2.2 Subjected norm

Subjective norms refer to the perceived societal influence to indulge or not to indulge in specific conduct [14]. It comprises an effect on other people in society and motivation to comply with other people's views [18]. Family buying habits and choices significantly influence dairy product consumption [22]. Thus, subjective norms can play a decisive role in consumers purchasing dairy products [20,21]. Therefore, the study adopts the hypothesis of having the influence of subjective norms over the purchase intention of dairy products.

H2 Subjective norms are associated with the purchasing behavior of dairy products.

### 2.3 Perceived behavior control

Perceived behavioral control refers to an individual's perceived ease or difficulty in performing particular behavior [14]. According to the TPB model, developing perceived behavioral control before generating is essential [23]. The perceived affordances were perceptual cues consumers possessed and used to evaluate products before purchasing [24]. Many researchers have confirmed that confidence in the ability of an individual to control their behavior showed a positive relationship with purchase intention of dairy products [25,26]. Based on this discussion, we propose the below hypothesis.

H3 Perceived behavioral control is associated with the decision to buy dairy products.

### 2.4 Marketing mix

The marketing mix (product, price, place, and promotion) is the variable a marketer can manage to influence the buyer's response. We used a marketing mix to identify how this set of variables related to the different attributes of the TPB and how they affect consumers' purchase intentions of dairy products in Bangladesh. Consumers use other evaluation parameters when choosing suitable products to meet their consumption needs [27]. Consumer behavior literature suggests that consumers perceive a product as features such as product diversity, maintaining hygiene, and naturalness. Buying one product over another is primarily determined by the mixture of these features. Price is the most noteworthy feature that attracts consumers most [28]. Product quality is positively related to customer satisfaction [29,30]. Visual and informative elements on food packaging are frequently used to influence individuals of all ages. Price significantly affects consumers' purchase intention of any products or services. More amazing prices may not discourage customers since they consider the items or services to be of more excellent quality or status. Different studies found that price significantly influences consumer purchases of dairy products in Bangladesh [31,32].

On the other hand, price-sensitive purchasers may prioritize cost over other factors while making purchases [33]. The availability of a product can significantly impact consumer behavior, and it is regarded as one of the influencing factors of consumer behavior. Product availability affects consumer behavior in several dimensions. These are the consumer attitude towards the product's importance, the likelihood of substituting another product, consumer cultural level, and the general public's trust in the economic unit and product line. Marketing strategies are not implemented solely by achieving production goals, pricing, or promotional activities. They are also linked to a successful product distribution process, enabling the company to supply the goods in the proper location on time. Consumers' purchase intention gradually increases when they access the products.

In contrast, lack of supply negatively affects consumer intake of milk [34]. Advertising involves all marketing strategies used to inform, motivate, and recall potential customers regarding the products or services of a company [35]. Promotion means communicating with and convincing potential customers to purchase the products by recognizing their needs and requirements. Promotional activities include advertising, promotional events, personal selling, etc. All these components combine to make the promotion mix fulfill the marketing goals. In consumer marketing, the insignificance of promotional activities could emerge if consumers are suspicious of the promotional activity. To be successful in promotional approaches, consumers need to be aware of and utilize the promotional strategies that motivate their purchasing intention. Again, information can influence buying behavior, but products must satisfy customer expectations. Therefore, companies could better market their products with the appropriate promotional activities and attract prospective consumers to purchase products. Here, we can propose the following hypotheses:

H4 Marketing mix associated with purchasing intention of dairy products.

H5 The marketing mix is related to attitude towards behavior.

H6 The marketing mix is related to subjective norm.

H7 The marketing mix is related to perceived behavioral control.

## 3 Materials and methods

### 3.1 Sample and sampling technique

The survey was done in Bangladesh in 2022, encompassing all areas. The target audience was individuals over 18 who purchased dairy products. A nationwide online survey with a structured questionnaire utilizing Google forms was designed for the primary data collection. Respondents were invited to participate in the survey via social media and personalized emails containing a link to the study. Convenience sampling technique was used to collect the data from the respondents. There were 319 replies received, where the number of incomplete responses was nine (9). After removing invalid responses, 310 legitimate responses were evaluated for analysis.

### 3.2 Questionnaire design

A structured questionnaire was prepared to collect necessary data about how the marketing mix influences consumers' purchasing decisions for dairy products with the Theory of Planned Behavior framework. The questionnaire is divided into four main parts. There were multiple items from existing literature [16,36], and some self-generated items were used to develop the questionnaire. The opening part was aimed at informing respondents about the goal of the survey, how they should complete the questionnaire, and the researcher's contact information, and to thank them for their participation before they began filling out the questionnaire. Informed consent was obtained from all participants about the purpose of the research. It also assured respondents of their privacy while participating in the study and that participation was optional, and those respondents could opt out at any time without penalty. The respondents agreed or disagreed with the declarations by clicking on the response category to respond.

The questionnaire starts with some background information. The questions include whether the respondents go to the market to buy dairy products, what they buy from the market, for which they buy them, how often they go to the market to purchase dairy products, and the distance to the market. The second section includes consumers' knowledge about the marketing mix. A brief description of the marketing mix is provided so that consumers can easily understand the marketing mix's concept and feel accessible to the response. The third section consists of the Theory of Planned Behavior (TPB). Here, all the TPB constructs also start with a short description to better understand the respondents. The following section includes the socio-demographic information of the respondents. This section addressed the questions regarding gender, age, income, the proportion of income spent on dairy products, level of education, working status, marital status, residential area, family size, and the number of children in the family. The demographic questions were used to create a profile of the study's sample of respondents. The questions were designed following a combination of open-ended and closed-ended questioning patterns. The questionnaire was pre-tested before launching to minimize unnecessary information. Respondents were requested to comment on the construct items' comprehensibility, context-specific relevance, question sequencing, and time management. The questionnaire was revised following minor corrections, modifications, and adjustments and finally sent to the respondents.

### 3.3 Data analysis

Descriptive and multivariate analyses were used to analyze the data, including exploratory factor analysis, reliability analysis, structural equation modeling (SEM), and multiple regression analysis.

#### 3.3.1 Exploratory factor analysis

This study used exploratory factor analysis to investigate the validity and uncover the underlying structure of variables. Factor loading is one of the principal criteria for analyzing the exploratory factor. It illustrates a close correlation between the variable and the common factor, illustrating a comparative degree of observed variables and a common factor above 0.3.

#### 3.3.2 Reliability analysis

Cronbach's alpha is a widely used method for determining the internal consistency of each latent variable, i.e., how strongly items in the construct are linked. Cronbach alpha to assess internal consistency can be classified as excellent (α ≥ 0.9), good (0.7 ≤ α < 0.9), acceptable (0.6 ≤ α < 0.7), poor (0.5 ≤ α < 0.6), and unacceptable (α < 0.5) [37].

#### 3.3.3 Structural equation modeling

Structural Equation Modeling and Maximum Likelihood (ML) procedures were estimated using SPSS AMOS, version 23, software to examine the latent variables within their causal structure. Utilizing the comparative fit index (CFI), the goodness of fit index (GFI), and the adjusted goodness of fit index (AGFI), the adequacy of the model was determined [38]. The normed chi-square index (chi-square/df) should be less than 3 [39], but the normed fit index (NFI) and incremental fit index (IFI) should be greater than 0.9 [38]. The parsimony-adjusted normed fit index (PNFI) and comparative fit index (PCFI) should be greater than 0.5 [40]. Lastly, the root-mean-square error of approximation (RMSEA) should be less than 0.08 [40] was used to evaluate the adequacy of the model.

## 4 Results

### 4.1 Socio-demographic profile of the respondents

Table 1 summarizes the socio-demographic characteristics of the respondents. In the case of gender ratio, the result showed that among 310 respondents, males (78.7%) are higher than females (21.3%). Most respondents (64.2%) aged between 19 and 30 years were considered young, followed by 24.8% of respondents aged between 31 and 45, regarded as middle age. About 11% of the total respondents were aged 46–60 years and considered older citizens. Regarding marital status, 58.7% were unmarried, and 41.3% were married. The education level shows most respondents were graduates (75.5%), whereas non-graduates were 24.5%. The employment status indicated that government and private service holders were equal (25.5%), and the unemployed was 49.0%. Regarding income, 48.1% of respondents earned BDT^1^ 5000–25000 per month, 40.0% earned between BDT 26000–55000 per month, and 11.9% earned between BDT 56000 above 76000 months. Almost one-third of families (70.6%) belonged to extended families, whereas the rest of the participants (29.4%) belonged to nuclear families. Most (58.7%) of respondents had no children in their families, whereas 41.3% had one or more children, as most were unmarried. In the case of residential areas, 35.7% of the total respondents were from the West region, followed by the North region 27.3%, the East region 22.5%, and the South region 14.1%.

**Table 1:** Socio-demographic characteristics of the respondents.

Variables	Description	Frequency	Percent (%)
Gender	**Gender of the respondents**		
	Male	244	78.7
	Female	66	21.3
Age	**Age of the respondents (in years)**		
	Young (19 to 30)	199	64.2
	Middle age (31 to 45)	77	24.8
	Elder (46 to 60)	34	11.0
Marital status	**Marital status of the respondents**		
	Married	128	41.3
	Unmarried	182	58.7
Education	**Highest educational level of the respondents**		
	Graduates	234	75.5
	Non-graduates	76	24.5
Profession	**Profession of the respondents**		
	Government service holder	79	25.5
	Private service holder	79	25.5
	Unemployed	152	49.0
Income	**Income of the respondent's (currency BDT)**		
	5000–25000	149	48.1
	26000–55000	124	40.0
	55000 and above 76000	37	11.9
Family size	**Number of members in a family**		
	Nuclear family (e.g., a couple and their dependent children)	91	29.4
	Extended family (family including grandparents, aunts, uncles, and other relatives)	219	70.6
Number of children	**Number of children in a family**		
	No children	182	58.7
	One and more children	128	41.3
Residing location	**Residences of the respondents**		
	North	85	27.3
	South	44	14.1
	East	70	22.5
	West	111	35.7

### 4.2 Dairy products purchase-related information

The dairy items purchased from the market were divided into two categories (milk and milk products). Table 2 shows that the majority (82.3%) of respondents buy fresh milk from the market, followed by powder milk (71.9%) and condensed milk (28.4%). Regarding milk products, sweets were purchased by 70.6% of respondents, followed by yogurt (61.3%), butter (26.5%), and cheese (19%). Only 1% of respondents buy others (i.e., milkshakes and *matha*^2^). Most of the respondents (89%) purchase dairy products for their own consumption, followed by parents (47.1%), children (36.8%), and family members (brothers, sisters, spouses, etc.) (17.1%). The table also shows that consumers buy dairy products (1.9%) for their relatives. About 41% of respondents visit the market weekly to purchase dairy products. About 26.0% of respondents visit twice weekly, followed by 9.35% three times a week and 8.06% more than three times. The table also shows that 14.84% of respondents visited the market daily to buy dairy products, while 1.29% of respondents did not visit the market at all to purchase dairy products. Most respondents (44.19%) spent their income less than or equal to 5% buying dairy products. 32.90% of respondents spent 6–10% income purchasing dairy products, followed by 20.97% who spent 11–15%. Only 1.94% of respondents spent more than 16–20% of their income purchasing dairy products.

**Table 2:** Dairy products purchase-related information.

Dairy products	Respondents (Number)	Percent (%)
Fresh milk	255	82.3
Powder milk	223	71.9
Condensed milk	88	28.4
Butter	82	26.5
Cheese	59	19.0
Sweet	219	70.6
Yogurt	190	61.3
Other	3	1.0
**Consumers**		
Own consumption	276	89.0
Parents	146	47.1
Children	114	36.8
Family members	53	17.1
Relatives	6	1.9
**Frequency of purchase**		
Everyday	46	14.84
Once/week	127	40.97
Twice/week	79	25.48
Three times/week	29	9.35
More than three times/week	25	8.06
Never	4	1.29
**Income spent**		
Less than or equal to 5%	137	44.19
6–10%	102	32.90
11–15%	65	20.97
16–20%	6	1.94

### 4.3 Measure validation

Exploratory factor analysis was used to examine consumer purchase decisions, identify the variables that underpin them, and empirically assess the resulting measures' one-dimensionality. The Kaiser-Mayer-Olin (KMO) and Bartlett's sphericity tests evaluated the data's fitness before factor analysis. The KMO sample adequacy value is 0.882, greater than the suggested cutoff value of 0.60 [40]. The inter-item correlations were substantial enough for Principal Component Analysis (PCA) (chi-square = 4894.38, df = 253, p < 0.05) [40]. Communalities measure the percentage of variance explained by all (retained) components. Communality levels of less than 0.3 are usually considered low. The lowest value reported in this investigation was 0.70, which is strong enough. The initial eigenvalues provide information for each factor solution. According to this rule, every factor needs to be kept with an eigenvalue greater than one. The result reveals that an eight-factor solution best fits which accounts for 82.01% of the total variance explained, with the total variance explained at 65.8%. The PCA with varimax rotation condensed many variables into a small set that retains most data from the more extensive group. Following that, one item for the product, one for the price, one for the place, one for promotion, one for subjective norm, and three items for perceived behavioral control were deleted. The following elements have a high loading on their associated factors, ranging from 0.703 to 0.901 (see Table 3). Before calculating the extracted Cronbach's alphas, composite reliabilities, and average variance, the researchers ran separate studies for each element. The different component values indicate that they were appropriately assessed. In this investigation, all Cronbach's alphas are above the recommended criterion of 0.7 (see Table 4). Coefficients were significantly different from zero, and loadings between latent and observed variables were consistently high (>0.7). Consequently, the latent variables adequately characterize the observed variables [40]. Specifically, the factor's composite Reliability (R.C.) and average variance extracted (AVE) exceeded the reference criterion of 0.7 and 0.5, respectively (see Table 4). Harman's single factor has been employed for determining common method bias (CMB). Harman's single factor test for this study was 36.93%, less than 50%, which means there is no common method bias.

**Table 3:** Descriptive statistics and factor loading of the scale items (Scale ranges from 1 to 7).

Items	Statement	Factor Loading	Mean	Std. Deviation
Product_1	The dairy products that you choose must be of good quality	0.809	6.09	1.317
Product_2	The dairy products that you choose must be environment friendly	0.802	5.92	1.404
Product_3	The dairy products that you buy must be labeled properly	0.786	6.01	1.507
Price_1	The chosen dairy products price is high	0.798	5.12	1.496
Price_2	The chosen dairy products price is reasonable	0.828	5.09	1.520
Price_3	The chosen dairy products price is negotiable	0.703	5.57	1.419
Place_1	Readily available to purchase	0.837	5.56	1.437
Place_2	Easily accessible in the outlet stores	0.812	5.70	1.341
Place_3	Convenient to get at any location without disruption of supply chain	0.807	5.15	1.669
Promotion_1	The dairy products that you choose must have an attractive advertising program	0.897	4.79	1.482
Promotion_2	The dairy products that you choose must often have attractive promotional programs	0.877	4.93	1.470
Attitude_1	The purchase of the dairy products in the next month will	0.828	5.39	1.049
Attitude_2	The purchase of the dairy products in the next month will	0.852	5.39	1.126
Attitude_3	The purchase of the dairy products in the next month will	0.802	5.37	1.155
Attitude_4	The purchase of the dairy products in the next month will	0.739	5.43	1.228
SN_1	People who are important to me think I should purchase dairy products over the next month	0.833	5.08	1.344
SN_2	People who are important to me approve of my dairy products purchase over the next month	0.857	5.17	1.304
SN_3	People who are important to me want me to purchase dairy products over the next month	0.807	5.18	1.403
PBC_1	Whether or not I purchase dairy products over the next month is entirely up to me	0.901	5.84	1.269
PBC_2	How much personal control do you feel you have over dairy products purchased in	0.868	5.67	1.259
INT_1	I intend to purchase dairy products over the next month	0.847	5.45	1.376
INT_2	I plan to purchase dairy products over the next month	0.844	5.49	1.376
INT_3	I want to purchase dairy products over the next month	0.810	5.52	1.427

**Table 4:** Validity and internal consistency reliability.

Constructs	Items	Cronbach's Alpha	Composite Reliability (R.C.)	Average Variance Extracted (AVE)
Marketing mix	Product_1	0.872	0.956	0.665
	Product_2			
	Product_3			
	Price_1			
	Price_2			
	Price_3			
	Place_1			
	Place_2			
	Place_3			
	Promotion_1			
	Promotion_2			
Attitude	Attitude_1	0.883	0.881	0.650
	Attitude_2			
	Attitude_3			
	Attitude_4			
Subjective norm	SN_1	0.907	0.871	0.693
	SN_2			
	SN_3			
Perceived behavioral control	PBC_1	0.793	0.878	0.783
	PBC_2			
Intention	INT_1	0.958	0.873	0.696
	INT_2			
	INT_3			

### 4.4 Structural analysis and model testing

**Fig. 1:**
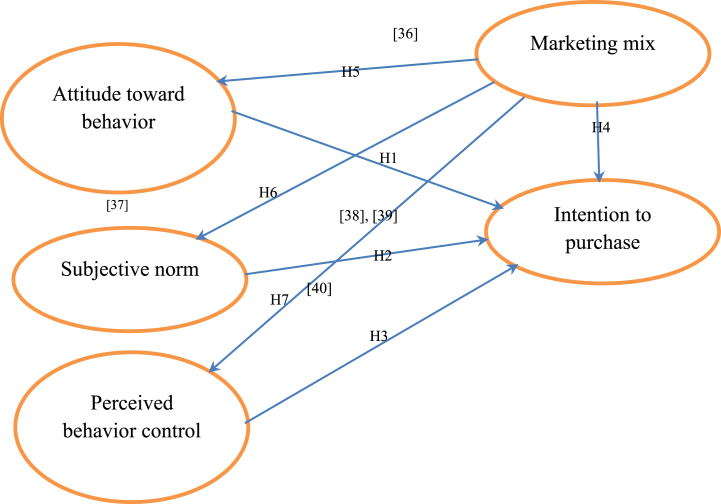
Conceptual framework.

**Fig. 2:**
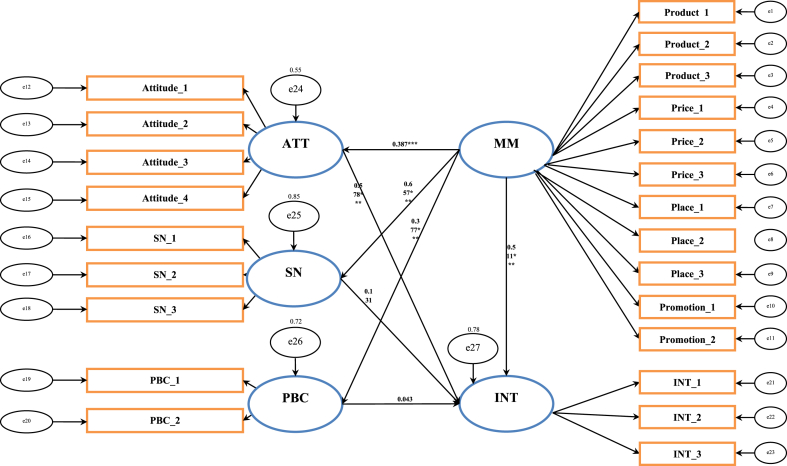
Path Analysis: Results of the structural equation modeling (Source: Authors estimation, 2022).

The analysis is based on the assumption that the data are normal Fig. 1. A series of indicators must be examined in SEM to evaluate the model's fit. The goodness of fit measures for the structural model exhibited values within acceptable ranges (CMIN/DF = 1.802; GFI = 0.904; AGFI = 0.875). TLI = 0.958, and CFI = 0.964; NFI = 0.924 and RMSEA was determined to be 0.051. The model's standardized estimates are displayed in Fig. 2. The significant path coefficients (p ≤ 0.05) support the presented model. The evaluation of the proposed hypotheses, based on the analysis of the SEM results, is presented in Table 5.

(Source: Modified and adopted, Karambut, 2021).

**Table 5:** Hypothesis testing.

Hypothesis				Standardized regression weight	C.R.	P value	Decision
H_1_	INT	<---	ATT	0.578	6.211	***	Accepted
H_2_	INT	<---	SN	0.131	1.798	0.072	Rejected
H_3_	INT	<---	PBC	0.043	0.590	0.555	Rejected
H_4_	INT	<---	MM	0.511	5.452	***	Accepted
H_5_	ATT	<---	MM	0.387	6.719	***	Accepted
H_6_	SN	<---	MM	0.657	8.494	***	Accepted
H_7_	PBC	<---	MM	0.377	4.817	***	Accepted

***p ≤ 0.01.

#### 4.4.1 Path Analysis

After gaining the appropriateness of the measurement, the SEM analysis and hypotheses testing was performed, and the results were listed in Fig. 2 and Table 5, respectively. The hypotheses were tested based on the standardized estimates of the structural equation modeling. Concerning H_1_, we found a strong positive and significant relationship between the attitude towards behavior and intention to purchase dairy products (β=0.578;p≤0.01), indicating H_1_ is supported. Therefore, people are more prone to buy dairy products based on their attitudes toward dairy products. On the contrary, the path coefficient of 0.131 for subjective norms and intention to purchase on dairy products is not significant. Thus, H_2_ is not supported. The results suggested that the societal influence doesn't bother the purchasing intention of dairy products instead the own demand does. Similarly, the study found no significant relationship between perceived behavioral control and intention to purchase dairy products. Hence the hypothesis (H_3_) that perceived behavioral control strongly influences the buying decision of dairy products is not accepted. The results highlighted that the purchase intention of dairy products is highly influenced by the marketing mix (product, price, place, and promotion); further, in the case of the structural link from marketing mix towards intention to purchase dairy products (***β*** = 0.511; *p* ≤ 0.01), the study found a strong positive and meaningful relationship which is significant at a 1% level. Therefore, hypothesis H_4_ is supported. In addition, the association proposed in H_5_ is confirmed; that is, the marketing mix influences the attitudes towards the behavior of purchasing dairy products (β=0.387;p≤0.01). The study demonstrated that marketing mix is a determining factor in influencing consumer attitudes. The results also provide strong evidence of the effect of marketing mix on the subjective norm (β=0.657;p≤0.01), therefore, H_6_ is accepted. This study's marketing mix had the most substantial influence on subjective norms. In favor of H_7_, the impact of marketing mix on perceived behavioral control is positive and significant at a 1% level (β=0.377;p≤0.01). Though there is no significant impact of perceived behavioral control and subjective norm on intention to purchase dairy products, a strong positive and meaningful relationship was found in the link of marketing mix on perceived behavioral control and subjective norm. Therefore, the marketing mix could influence personal control over purchasing dairy products. The marketing mix influenced the intention to purchase dairy products, attitudes towards behavior, subjective norm, and perceived behavior control.

## 5 Discussion

This study explored the factors influencing Bangladeshi consumers' intentions to purchase dairy products using the theory of planned behavior and marketing mix. We hypothesized that several constructs were associated with choice behavior based on TPB. The first hypothesis showed a positive association between attitude and dairy product purchasing. We discovered that attitude had a large, positive, and statistically significant influence on the likelihood of purchasing dairy products. Previous research has shown that attitude is an important predictor of behavioral intention [13,14]. The health benefits of dairy products are broadly acknowledged. People in Bangladesh typically purchase dairy products because of their nutritious value. The subjective norm had no significant impact on the purchase of dairy products. The result supports previous findings [41,42]. This result demonstrates that subjective norms and social groupings couldn't impact how they choose dairy products. This result can be concluded that the purchasing behavior of dairy products has not yet become a social norm among the people of Bangladesh. This finding suggests that there is still a scope of leveraging the opinion leaders such as celebrities to stimulate intentions to buy dairy products. The perceived behavioral control had no significant impact on purchase intentions. This result indicates that, in Bangladesh, milk deficiency exists to meet the total demand of consumers. People's perceptions of behavioral control are influenced by their views about the availability of resources, necessary opportunities to achieve behavior, and how these resources and chances support behavior. The purchasing intention, attitudes towards purchasing behavior, subjective norm, and perceived behavior control were influenced by the marketing mix (product, price, place, and promotion) in this study. Product characteristics and product attributes determine whether people will purchase or not. When consumers buy a product or service to meet their requirements, they believe in the advantages instead of the thing itself [43]. Based on the result of the factor loading, product quality (0.809) was the most important determinant that affected consumer purchase intention. On the other hand, many of today's value-conscious buyers may purchase dairy products based on price rather than other considerations. This study identified the reasonable price (factor loading, 0.828) as the primary indicator of consumer purchase intention of dairy products. On the contrary, high milk price is a barrier to milk intake, particularly among lower socio-economic groups [33]. Promotion is a technique for bringing a product to the notice of potential customers. Advertising and price discounts are two effective promotional strategies that directly impact purchasing decisions [44]. Much research has been undertaken to understand better and investigate how product image/packaging affects customer behaviors such as buy intent, satisfaction, and loyalty and found a significant relationship [45,46]. Among different items, advertising programs (0.897) were the most vital parameter influencing consumers to purchase dairy products. The study also found that dairy product availability (0.832) was the significant determinant of consumer purchase intentions. The result is consistent with the previous study on consumers' purchase intention of dairy products [34].

The study's limitations were that a relatively small number of people were interviewed, those with a better internet connection and electronic devices. Respondents' truthfulness in answering questions was not checked, and the influence of family members and peers on the interviewees may have caused subjective bias. The study proposed increasing the number of rural respondents better to describe Bangladeshi consumers' behavior towards products.

## 6 Conclusions

The dairy industry is one of the country's most important industries, with enormous potential for economic development. Although there is a lot of research conducted on consumer' purchase intentions regarding dairy products, to our knowledge, no systematic study has been conducted using the theory of planned behavior with the marketing mix. This study examined consumers' purchase intentions toward dairy products in Bangladesh. Structural equation modeling was used to identify the factors that influence consumer purchase intention for dairy products. The results showed that marketing mix and attitude positively and significantly influenced the consumer's purchase intention of dairy products. In addition, the marketing mix has a significant influence on attitude towards behavior, subjective norm, and perceived behavioral control. However, the study found no significant relationship between perceived behavioral control and subjective norm and purchase intention. The results can be beneficial for marketing companies to develop a better product, reasonable pricing, promotional strategies, and correct placement that will attract consumers' intention to purchase dairy products. Additionally, the research findings can assist dairy manufacturing companies in focusing on consumer preferences and developing new products that will succeed in the future market. The study's outcome suggests that increasing the availability of milk and dairy products in Bangladesh could help improve consumers' purchase intentions. Consumers' perceived behavioral control might be increased by encouraging them to eat more dairy items by swapping milk for other beverages and improving their cooking skills with dairy products. Also, the practitioners should emphasize both the nutritional and practical benefits to boost the favorable attitude of the consumers toward dairy products.

## Author contribution statement

Md. Shaikh Farid: Conceived and designed the experiments; Performed the experiments; Analyzed and interpreted the data; Contributed reagents; Materials, Analysis tools or data; Wrote the paper.

Alessio Cavicchi: Conceived and designed the experiments; Analyzed and interpreted the data; Contributed reagents.

Md. Rasheduzzaman: Analyzed and interpreted the data; Wrote the paper.

Md. Mostafizur Rahman; Swarup Barua; Dilshad Zahan Ethen, Dabasis Sharma and Fardous Ara Happy: Analyzed and interpreted the data; Contributed reagents; Materials, Analysis tools or data.

Mohammed Jahangir Alam: Analyzed and interpreted the data; wrote the paper.

## Data availability statement

Data will be made available on request.

## Ethics approval and consent to participate

Not applicable

## Consent for publication

Not applicable

## Funding

This research did not receive any specific grant from public, commercial, or not-for-profit funding agencies.

## Declaration of competing interest

The authors declare that they have no known competing financial interests or personal relationships that could have appeared to influence the work reported in this paper.

## Footnotes

1 Bangladesh Taka (1 USD = 107.40 Tk. Source: https://www.bb.org.bd/en/index.php/econdata/exchangerat).

2 Special drink made of milk, ice, and sugar.
