# Supplementary material for: Assessment of marketing mix associated with consumer's purchase intention of dairy products in Bangladesh: Application of an extended theory of planned behavior
Source: Heliyon. 2023 May 30;9(6):e16657. doi: 10.1016/j.heliyon.2023.e16657 (PMC10250725; doi:10.1016/j.heliyon.2023.e16657)
Supplement: Multimedia component 1 [file mmc1.pdf]

Survey Questionnaire

The survey is a part of a university (Universita di Pisa) thesis; it aims to Assessing marketing mix influence on buying decision of dairy products: an exploratory analysis by using the Extended Theory of Planned Behavior. The survey consists of 4 sections. Data collection is entirely voluntary. Please feel at ease. The results of the survey will be scientifically analyzed and all findings will be compiled in the thesis.

Confidentiality of data
Data and results will be treated with the utmost confidentiality guaranteeing the anonymity of respondents.

Duration
The response time to complete the questionnaire should take approximately 8-10 **minutes**.

Contact information
If you have any questions, please contact: m.farid@studenti.unipi.it

Thank you in advance for your valuable response!

Md. Shaikh Farid, International Master of Science in Rural Development (IMRD), (Dept. Veterinary Science, Dept. Agriculture, Food and Environment and Dept. of Economics and Management), Pisa University, Italy.

* Required

1. **Consent to participate:** I herewith confirm that I have read and understood the above information. I am at least 18 years old and give my consent to participate in this research. *

Mark only one oval.

- Yes
- No

Section I

Background Information

2. Do you buy dairy products from the market?

Mark only one oval.

- Yes
- No

3. What are the dairy products you buy from the market?

Check all that apply.

- [ ] Fresh milk
- [ ] Powder milk
- [ ] Condensed milk
- [ ] Butter
- [ ] Cheese
- [ ] Sweet
- [ ] Yoghurt

Other: [ ] ______________________

4. For whom you buy the dairy products?

Check all that apply.

- [ ] Myself
- [ ] Parents
- [ ] Children

Other: [ ] ______________________

5. What is the distance of the market from your residence in kilometer?

____

6. On an average, how often do you go to market to buy dairy products?

Mark only one oval.

- Everyday
- Once/week
- Twice/ week
- Three times/week
- More than three times/ week
- Never

BEFORE GOING TO NEXT SECTION, PLEASE TAKE A FEW MINUTES TO READ THE FOLLOWING PARAGRAPH

Section II: Marketing Mix

A marketing mix includes multiple areas of focus as part of a comprehensive marketing plan. The term often refers to a common classification that began as the four Ps: product, price, place, and promotion.

Please tick one box for each statement below to what extent do you agree or disagree

1- Strongly Disagree
2- Disagree
3- Slightly Disagree
4- Neutral
5- Slightly Agree
6- Agree
7- Strongly agree

Product: In marketing, a product is an object, or system, or service made available for consumer use as of the consumer demand; it is anything that can be offered to a market to satisfy the desire or need of a customer. In retailing, products are often referred to as merchandise, and in manufacturing, products are bought as raw materials and then sold as finished goods. A service is also regarded as a type of product. (Dairy products include milk, butter, cheese, yoghurt etc.)

7. To what extent do you agree or disagree with the following statements? *

Mark only one oval per row.

	1- Strongly Disagree	2- Disagree	3- Slightly Disagree	4- Neutral	5- Slightly Agree	6- Agree	7- Strongly agree
The dairy products that you choose must be of good quality							
The dairy products that you choose must be diverse in type							
The dairy products that you choose must be environment friendly							
The dairy products that you buy must be labelled properly							

Price: Price in marketing mix refers to the value we pay in exchange for the product and services offered by a company.

8. To what extent do you agree or disagree with the following statements? *

Mark only one oval per row.

	1- Strongly Disagree	2- Disagree	3- Slightly Disagree	4- Neutral	5- Slightly Agree	6- Agree	7- Strongly agree
The chosen dairy products price is expensive	◯	◯	◯	◯	◯	◯	◯
The chosen dairy products price is reasonable	◯	◯	◯	◯	◯	◯	◯
Then chosen dairy products price is negotiable	◯	◯	◯	◯	◯	◯	◯
The bulk dairy product price must be lower than retail price	◯	◯	◯	◯	◯	◯	◯

Place: Place in marketing mix refers to the geographical location in which the company sells its products and provides its services.

9. To what extent do you agree or disagree with the following statements? *

Mark only one oval per row.

	1- Strongly Disagree	2- Disagree	3- Slightly Disagree	4- Neutral	5- Slightly Agree	6- Agree	7- Strongly agree
Readily available to purchase	⬭	⬭	⬭	⬭	⬭	⬭	⬭
Easily accessible in the outlet stores	⬭	⬭	⬭	⬭	⬭	⬭	⬭
Convenient to get at any location without disruption of supply chain	⬭	⬭	⬭	⬭	⬭	⬭	⬭
Possible to order via online	⬭	⬭	⬭	⬭	⬭	⬭	⬭

Promotion: Promotion in the marketing mix refers to the communication that aims at promoting a product, activity, or a brand among the target customers; in order to drive sales, and involves both buyer and seller. Advertising is the action of calling public attention to an idea, good, or service through paid announcements by an identified sponsor.

10. To what extent do you agree or disagree with the following statements? *

Mark only one oval per row.

	1- Strongly Disagree	2- Disagree	3- Slightly Disagree	4- Neutral	5- Slightly Agree	6- Agree	7- Strongly agree
The dairy products that you choose must have attractive advertising programme	⬭	⬭	⬭	⬭	⬭	⬭	⬭
The dairy products that you choose must often have attractive promotional programmes	⬭	⬭	⬭	⬭	⬭	⬭	⬭
The dairy products that you choose must often have buy one get one free offer	⬭	⬭	⬭	⬭	⬭	⬭	⬭

Section III: Theory of Planned Behavior (TPB)

BEFORE GOING TO NEXT SECTION, PLEASE TAKE A FEW MINUTES TO READ THE FOLLOWING PARAGRAPH

The theory of planned behavior proposes three fundamentally separate drivers of intention. These are attitude toward the behavior, subjective norm, and perceived behavioral control.

Attitude: Attitude toward the behavior refers to the degree to which a person has a favorable or unfavorable judgment or appraisal of behavior.

11. The purchase of the dairy products in the next month will *

Mark only one oval.

	1	2	3	4	5	6	7	
Very bad	⬭	⬭	⬭	⬭	⬭	⬭	⬭	Very good

12. The purchase of the dairy products in the next month will *

Mark only one oval.

	1	2	3	4	5	6	7	
Very unpleasant	⬭	⬭	⬭	⬭	⬭	⬭	⬭	Very Pleasant

13. The purchase of the dairy products in the next month will *

Mark only one oval.

	1	2	3	4	5	6	7	
Very much unenjoyable	⬭	⬭	⬭	⬭	⬭	⬭	⬭	Very much enjoyable

14. The purchase of the dairy products in the next month will *

Mark only one oval.

	1	2	3	4	5	6	7	
Extremely harmful	⬭	⬭	⬭	⬭	⬭	⬭	⬭	Extremely beneficial

Subjective norm: Subjective or social norms refer to the perceived social pressure to perform or not perform the behavior.

Please tick one box for each statement below

15. To what extent do you agree or disagree of the following statements? *

Mark only one oval per row.

	1- Strongly Disagree	2- Disagree	3- Slightly Disagree	4- Neutral	5- Slightly Agree	6- Agree	7- Strongly agree
People who are important to me think I should purchase dairy products over the next month							
People who are important to me approve of my dairy products purchase over the next month							
People who are important to me want me to purchase dairy products over the next month							
I feel under social pressure to purchase dairy products over the next month							

Perceived behavioral control: Perceived behavioral control (PBC) is one of the most important factors influencing consumer's behavior. Perceived behavioral control is the people's perception of the ease or difficulty of performing the behavior of interest.

Please tick one box for each statement below

16. Whether or not I purchase dairy products over the next month is entirely up to me. *

Mark only one oval.

	1	2	3	4	5	6	7	
Strongly disagree								Strongly agree

17. How much personal control do you feel you have over dairy products purchase in the next month? *

Mark only one oval.

	1	2	3	4	5	6	7	
Very little control								Complete control

18. To what extent do you feel that whether you purchase dairy products in the next month is beyond your control?

Mark only one oval.

	1	2	3	4	5	6	7	
Not at all								Very much so

19. I believe I have the ability to purchase dairy products in the next month *

Mark only one oval.

	1	2	3	4	5	6	7	
Definitely do not	⬭	⬭	⬭	⬭	⬭	⬭	⬭	Definitely do

20. How confident are you that you will be able to purchase dairy products in the next month? *

Mark only one oval.

	1	2	3	4	5	6	7	
Very unsure	⬭	⬭	⬭	⬭	⬭	⬭	⬭	Very sure

Purchase intention: Intention is the individuals' motivation to engage in a particular behavior.

21. To what extent do you disagree-agree with the following statements? *

Mark only one oval per row.

	1- Strongly Disagree	2- Disagree	3- Slightly Disagree	4- Neutral	5- Slightly Agree	6- Agree	7- Strongly agree
I intend to purchase dairy products over the next month	◯	◯	◯	◯	◯	◯	◯
I plan to purchase dairy products over the next month	◯	◯	◯	◯	◯	◯	◯
I want to purchase dairy products over the next month	◯	◯	◯	◯	◯	◯	◯

Section IV : Socio-Demographic information

Please complete Section (IV) by putting (tick) in the appropriate box.

22. What is your Gender? *

Mark only one oval.

◯ Male

◯ Female

23. How old are you? (Years) *

____

24. Where are you from? *

Mark only one oval.

- Dhaka
- Sylhet
- Chittagong
- Rajsahi
- Khulna
- Barishal
- Mymensingh
- Rangpur
- Other: ______

25. What is your marital status? *

Mark only one oval.

- Married
- Unmarried
- Divorced
- Widow

26. What is your level of education? *

Mark only one oval.

- Primary
- Secondary
- Higher-secondary
- Bachelor
- Masters
- Phd
- Post-doctoral
- Other: ______________________

27. What is your professional/ working status? *

Mark only one oval.

- Student
- Homemaker
- Self- employed (e. g. business, shopkeeper)
- Govt. Service holder
- Employee (e. g. other than govt. employee)
- Employer (e. g. owner of any organization)
- Unemployed
- Retired
- Other: ______________________

28. What is your monthly income? (BDT) *

Mark only one oval.

- 5000-15000
- 16000-25000
- 26000-35000
- 36000-45000
- 46000-55000
- 56000-65000
- 66000-76000
- Above 76000

29. What proportion of your total income is spent for dairy products? *

Mark only one oval.

- ≤5%
- 6-10%
- 11-15%
- 16-20 %
- ≥20%

30. Number of members in your family? *

31. Number of children in your family? *

Thank You!

Please click on "Submit" to complete the survey. If you are interested in receiving a short summary of the results, please send an email to m.farid@studenti.unipi.it or mdshaikh.farid@ugent.be

This content is neither created nor endorsed by Google.

Google Forms
